# Sudden infant death syndrome: deletions of glutathione-S-transferase genes M1 and T1 and tobacco smoke exposure

**DOI:** 10.1007/s00414-021-02556-5

**Published:** 2021-05-01

**Authors:** Anthea Mawick, Heidi Pfeiffer, Marielle Vennemann

**Affiliations:** Institute of Legal Medicine, University Hospital of Münster, Röntgenstr. 23, 48149 Münster, Germany

**Keywords:** SIDS, Sudden infant death syndrome, Tobacco smoke exposure, Glutathion-S-transferase, GSTM1, GSTT1

## Abstract

In developed countries, sudden infant death syndrome (SIDS) is the leading cause of death in infants in their first year of life. The risk of SIDS is increased if parents smoked during pregnancy and in presence of the child. Glutathione S-transferases (*GSTs*) catalyse the conjugation of glutathione with electrophilic compounds and toxins, making them less reactive and easier to excrete. As a gene dose effect was observed for *GSTM1* and *GSTT1*, the aim of this study was to investigate whether there is a connection between homozygous or heterozygous gene deletions of *GSTM1* or *GSTT1* and the occurrence of SIDS. We found that heterozygous deletion of *GSTM1* occurred significantly more frequently in the SIDS case group compared to the control group. A homozygous deletion of *GSMT1* was slightly more frequently in the control group. A homozygous gene deletion of *GSTT1* showed no significant difference between the SIDS group and the control group. We also found that in the SIDS group, the number of victims that were exposed to cigarette smoke was significantly higher than the number of victims without cigarette smoke exposure and that the mean lifetime of children whose mothers smoked was shorter in comparison with non-smoking mothers. In SIDS cases with homozygous gene deletions of *GSTM1*, the median life span of children with tobacco smoke exposure was 60 days shorter than without smoke exposure. In conclusion, the absence of these two genes is not the only trigger for SIDS but could be a critical aspect of SIDS aetiology, particularly in SIDS cases with smoking parents.

## Introduction

Sudden infant death syndrome (SIDS) is defined as “the sudden death of an infant under one year of age which remains unexplained after a thorough investigation, including a complete autopsy, examination of the death scene, and review of the clinical history.” (San-Diego-definition) [1]. Sudden infant death is a cause of death in childhood whose risk can be reduced through preventive measures so that the occurrence of SIDS has become fairly rare, with an incidence of 0.22 cases per 1000 live births in 2013 [2]. The distinct decrease of children dying of SIDS can be attributed, *inter alia*, to the “Back-to-Sleep” Campaign that originated in 1994 and is nowadays known as the “Safe-to-Sleep” Campaign [3, 4]. This campaign was launched by a consortium of US health agencies to educate parents and caregivers about how to practice safe infant sleep and thereby reduce the risk of SIDS. The recommendations include a baby’s sleep environment and putting the baby on his or her back to sleep [5].

According to the “Triple-Risk-Model” by Filiano and Kinney [6], SIDS only occurs if the following three aspects coincide: (1) the child is in a critical phase of development, (2) the child is vulnerable (premature baby or children with genetical risk factors), and (3) the child is exposed to exogenous stressors (e.g., prone sleeping, the mother’s tobacco use or overheating). For a long time, smoking during pregnancy has been known as a risk factor for low birth weight, growth retardation, premature birth [7], stillbirth [8], and the occurrence of SIDS among others [7, 9, 10]. The risk of developing SIDS is approximately threefold higher if the mother smokes [11]. Approximately two-thirds of the SIDS cases could be prevented if both parents refrained from smoking [10], since it is not only the mother’s smoking behaviour during pregnancy that has an impact on developing SIDS. Instead, the occurrence of SIDS is also affected by the passive smoking of the child through the father or another person in the household [9, 10, 12]. Maternal smoking behaviour has an influence on the child’s time of death as well. The risk of an early death by SIDS is higher if the mother smokes [11], and the number of smoked cigarettes is known to have an impact on SIDS [9–11].

Numerous potential genetic risk factors for the occurrence of SIDS with regard to smoking exposure, such as gene deletions of *GSTM1*, *GSTT1*, variants of the *FMO3* gene, or *CYP1A1* polymorphisms, were discussed recently [13–15]. But the exact relationship between genetic risk factors and the influence of different stressors needs further investigation to be fully understood.

*GSTM1* and *GSTT1* are members of the glutathione S-transferase supergene family that contains at least 16 genes [16]. According to the *Atlas of Genetics and Cytogenetics in Oncology and Haematology*,* GSTM1* is part of a cluster of highly polymorphic *GSTM* genes located on chromosome 1p13.3; it is approximately 20 kb in length and contains eight exons (http://atlasgeneticsoncology.org/Genes/GC_GSTM1.html). *GSTT1* is approximately 8,5 kb in length and is located on chromosome 22q11.2 [17], approximately 50 kb away from *GSTT2* gene, with which it shares considerable sequence consistency and a gene structure of five exons (https://www.ncbi.nlm.nih.gov/gtr/genes/2952/). Both *GSTM1* and *GSTT1* encode enzymes that induce the body’s detoxication of ingredients from cigarette smoke [18], of which there are more than 4700 in total [19]. In phase II of the metabolism, glutathione S-transferases catalyse the conjugation of reduced glutathione with electrophile compounds like carcinogens and toxins from the environment. As a result, the cell is protected against xenobiotics and oxidative stress [20]. The detoxification through *GSTM1* and *GSTT1* is not limited to only a few substance classes but rather includes numerous xenobiotics [21, 22]. Furthermore, the glutathione S-transferase’s activity impacts on human erythrocytes, protecting them from cytogenetic toxicity [23].

Variants in the *GSTM1* and *GSTT1* genes are considered to be particularly critical in the development of SIDS [16], lung function deficits in children [24], and apparent life-threatening events (ALTE) [25].

A *GSTM1 *null allele (GSTM1*0/0) results from an unequal crossing over between two highly identical 4.2 kb, repeating sequences that flank the *GSTM1* gene, which results in a 15 kb gene deletion, that includes the whole *GSTM1* gene [26]. A similar process leads to a 54 kb *GSTT1* gene deletion [21] and the emergence of the *GSTT1* null allele (GSTT1*0/0) [27]. In Europe, about 50% of the population have a GSTM1*0/0 and about 20% have a GSTT1*0/0 genotype [22, 28]. A gene dosage effect has been observed for both genes, meaning that a homozygous deletion of these genes is accompanied by a reduced detoxication of xenobiotics due to lack of enzyme activity [22, 27, 29]. Sprenger et al. found that a GSTT1*0/0 genotype correlates with a non-conjugator phenotype. Reduced enzyme activity was found in GSTT*1/0 genotypes, while the GSTT*1/1 genotype was found to be associated with high activity [27]. These results coincide with the study of Bruhn et al. with a differentiation between highly and intermediately active individuals [29]. Rebbeck examined multiple molecular epidemiological studies regarding the *GSTM1* and *GSTT1* genotypes, respectively, and the emergence of cancer. He describes a reduced elimination of electrophilic carcinogens if GST enzymes were absent or deficient and found that these genes are involved in the aetiology of cancer at different sites [22].

Several studies investigated smoking as a risk factor for SIDS [10, 11, 30–32]; others analysed how gene deletions of *GSTM1* and *GSTT1* affect the occurrence of SIDS [14, 33]. Recently, Filonzi et al. [13] combined both aspects in their study and investigated the correlation between the occurrence of SIDS, smoke exposure of the child, and the genotypes of *GSTM1* and *GSTT1*, respectively. The GSTM1*0/0 genotype was observed three times more frequently in SIDS cases compared to controls. The relationship between the number of gene copies of *GSTM1* and/or *GSTT1* and SIDS was also examined in other studies. In contrast to the work of Filonzi et al., they found that 0/0 genotypes of *GSTM1* and *GSTT1* did not occur more frequently in SIDS victims than in controls and concluded that the identification of the genotype does not help to identify a population with an increased SIDS risk [14, 33].

The aim of our study is to investigate a possible connection between the occurrence of SIDS and the deletion of the *GSTM1* or *GSTT1* gene, taking into account the smoking behaviour of the mother and her partner during pregnancy and after birth. The underlying hypothesis is that reduced copy numbers of *GSTM1* and *GSTT1* lead to reduced enzyme activities, which in turn increases the risk of SIDS and/or decreases the lifespan of SIDS victims. To allow a more detailed analysis of this effect, our study aimed at distinguishing between homozygous and heterozygous gene deletions for the first time.

## Material and methods

### The sample collective

A total of 257 SIDS samples (formalin-fixed and paraffin-embedded lung tissue, frozen lung tissue) were analysed in this study. They originate from the GeSID study [34], and the local ethical committee approved the use of the samples. Exclusion criteria were death before the 8th day or after the 12th month after birth, cases in which death was expected due to existing illnesses, an unnatural cause of death, and inadequate knowledge of the German language by the parents, so that no declaration of consent could be obtained. The questionnaires that were handed out to the parents after the children’s deaths included questions about sociodemographic factors, sleeping situation, feeding of the child, and the parents’ smoking behaviour during pregnancy and after birth. In 46 SIDS cases, there is no information on the parents’ smoking behaviour available. In 83% of the remaining 203 SIDS cases, the children were exposed to cigarette smoke during pregnancy and/or after birth. Of the smoking mothers, 7.4% smoked heavily (at least 20 cigarettes a day) and 56.7% moderately (up to 20 cigarettes a day). In 36 SIDS cases, the mother did not smoke, but the children were still exposed to smoke through the father. The 168 control samples are oral mucosal abrasions, of which 94 were taken in the course of the GeSID study. The 94 control samples from the GeSID study were children matched by age, gender, and geographical region. The other control samples were collected as part of the current study and were donated by adults. Since genetic characteristics do not change during the lifetime of an individual, control samples from adults are suitable for this study. No information was available on the smoking behaviour of the parents.

The sample set corresponds to the typical characteristics of SIDS: 62% of the SIDS victims were male and 38% female. 48.8% died in the 2nd to 4th month after birth, and 60% died during the cold months. The mean age at the time of death was 137.52 days (9–358 days).

### DNA extraction and quality control

DNA extraction was carried out using a standard xylol deparaffination followed by standard phenol–chloroform extraction and Chelex extraction using 5% Chelex (BioRad, Feldkirchen, Germany) [35].

To determine the quality and quantity of DNA, a quantification was performed using the PowerQuant® System (Promega) in a 10 µL volume following a fully validated standard procedure of this laboratory.

### Assessing the copy number of GSTM1 and GSTT1

Copy number assessment followed a protocol described by Nørskov et al. [36] with some modifications:

To determine the genotypes of *GSTM1* and *GSTT1*, we used a Relative Real-Time Quantitative PCR (7500 Real-Time PCR System; HID Real-Time PCR Analysis Software v1.1; Applied Biosystems) and absolute quantification with automatic baseline setting and a threshold of 0. For the singleplex reactions, 2 µL DNA (with a DNA concentration of 0.55 to 127 ng/µL) were added to 8 µL PCR Mix, consisting of 5 µL Master Mix (TaqPath Pro Amp Master Mix, Applied Biosystems), 0.5 µL Assay Mix, and 2.5 µL H_2_O. The assay contained 2.5 µL probe (250 nmol/L), 9 µL of each primer (900 nmol/L), and 29.5 µL H_2_O. For duplex reactions, 2 µL DNA were mixed with 8 µL PCR Mix, consisting of 5 µL TaqPath Pro Amp Master Mix, 0.5 µL Assay Mix, 0.5 µL RNaseP Assay, and 2 µL H_2_O. Primer and probe sequences as well as their concentration were adopted from Nørskov et al. [36]. To normalize for variations in DNA input, the 20-fold concentrated and VIC- labelled TaqMan™ Copy Number Reference Assay “human, *RNaseP*” from Applied Biosystems was used. This internal control gene does not show any copy number variation but exhibits two gene copies in all samples. The relative quantity of the target genes compared to this normalizer allows the determination of copy numbers without influence of DNA input amounts or slight technical variation during sample processing.

We first examined 10 samples with an approximate DNA concentration of 50 ng/µL and performed a singleplex absolute quantification reaction. We chose one sample that contained at least one copy of *GSTM1* and one copy of *GSTT1* based on the Ct values for identifying the positive controls and for validation of the ΔΔCt method (see below). The Ct values for non-null samples were 22.7–23.4, while the Ct value for all null samples was 40. We quantified this sample again with 22 more samples and were hereby able to identify our 4 positive controls GSTM1*1/0 and *1/1 and GSTT1*1/0 and *1/1 by analysing the relative quantities based on differences in the Ct value. We confirmed the copy numbers of positive controls using the Human Random Control DNA Panel (Sigma Aldrich). It represents a control population of 480 UK Caucasian blood donors. Rose-Zerilli et al. [37] list two samples of this panel for *GSTM1* and *GSTT1* for two copy numbers, 1 copy number and 0 copy numbers, respectively. We used two of these samples with one copy for each, *GSTM1* and for *GSTT1* and two samples with two copies for validation by amplifying these samples and the previously identified positive controls on one plate and confirming identical relative Ct values. After that we performed the quantification in duplex reactions (*GSTM1* + *RNaseP* and *GSTT1* + *RNaseP*) for all samples to determine their copy number. All samples were examined in quadruplicates in 96-well plates. For each run, 4 negative controls were examined, as well as a positive control with GSTM1*1/1 and GSTM1*1/0 or GSTT1*1/1 and GSTT1*1/0, also in quadruplicate.

The number of gene copies was examined by using the ΔΔCt method, where Ct_(GST)_ – Ct_(RNaseP)_ = ΔCt and ΔCt_(GST)_ – ΔCt_(reference genotype)_ = ΔΔCt is. The relative quantity (RQ; 2^− ΔΔCt^) can be determined from the ΔΔCt value, which, multiplied by 2, represents the copy number [38].

The copy number of each individual sample was calculated and compared to the calculated copy number from the mean Ct value of the 4 replicates. Replicates of a sample with deviations in the Ct value of > 0.49 were tested again and excluded from further analysis if the deviation persisted. We evaluated the differences in ΔΔCt values between 1/1 and 1/0 genotypes and 2/1 and 1/0 genotypes, respectively. The difference between 1/1 and 1/0 should be − 1.00 (ΔΔCt_1/1_ – ΔΔCt_1/0_). The difference between 2/1 and 1/0 should be − 1.58 (ΔΔCt_2/1_ – ΔΔCt_1/0_). Since the ΔΔCt value indicates the relative difference in abundance of the samples compared to the calibrator sample, one can expect that the ΔΔCt value for one copy is 1, since the difference between sample and calibrator is 1: 2 copies. The expected ΔΔCt value for 2 copies is 0 because there is no difference to the calibrator sample. For 3 copies, the expected ΔΔCt is − 0.58 due to exponential growth; the difference between the sample and the calibrator sample is 2: 3. Accordingly, when comparing the ΔΔCt values between the genotype groups, one expects a value of − 1 for 1/0 vs. 1/1 (ratio 1: 2) and − 1.58 for 1/0 vs. 2/1 (ratio 1: 3). The closer the calculated values are to these values, the more precise is the assignment of the samples to a genotype group.

### Statistical analyses

The Chi^2^ test was used to evaluate the mother’s and father’s smoking behaviour and its influence on the children’s lifetime. The Chi^2^ test was also used to compare the genotype frequencies in the SIDS case and control cohorts.

The statistic evaluation of the smoking behaviour with regard to non-smoking and smoking parents was conducted with the exact two-sided binomial test.

The Kruskal–Wallis test was used to determine the influence of smoking behaviour and genes on the children’s lifetime.

IBM SPSS version 25 was used to carry out the calculations.

### Validation of the ΔΔCt method and plate-to-plate-reproducibility

The validation procedure followed a protocol described by Nørskov et al. [36]:

Since the use of the ΔΔCt method requires, among other things, the amplification efficiencies of the GST genes and the *RNaseP* reference genes to be approximately the same and close to 100% for singleplex reactions and multiplex reactions [36], a sample with a 1/0 genotype was established for *GSTM1* and *GSTT1* to create a standard curve to determine the amplification efficiencies. Standard curves were developed for both genes in singleplex and duplex reactions, and the amplification efficiencies in the duplex reactions were compared with the GST gene and *RNaseP*. The amplification efficiency is calculated from the slope of the standard curve using the term E = (10^(−1/slope)^ -1), where the Ct values are plotted against log DNA concentrations. A slope of 0 indicates the same amplification efficiency of the two examined genes. Slopes of less than ± 0,1 can be accepted for a use of the ΔΔCt method [36]. A total of 6 standard DNA concentrations with threefold dilution were measured in triplicates in duplex reactions, starting at 50 ng. For this purpose, 2 µL DNA was added to 8 µL PCR Mix, consisting of 5 µL TaqPath Pro Amp Master Mix, 0.5 µL Assay Mix, 0.5 µL RNaseP Assay, and 2 µL H_2_O. The concentrations for primers and probes were adopted from Nørskov et al. [36].

After validation of the method, the copy numbers of *GSTM1* and *GSTT1* of the SIDS cases and the controls were determined. As no clear results could be achieved with 4 SIDS samples, these samples were excluded from further analyses. In order to determine the reproducibility between quantification plates, the mean Ct, ΔCt, and ΔΔCt values of the plates for the 1/0, 1/1, and 2/1 genotypes were compared with one another. A 1/0 genotype corresponds to 1 gene copy, a 1/1 genotype corresponds to 2 gene copies, and a 2/1 genotype corresponds to 3 gene copies.

## Results and discussion

### Validation and reproducibility of copy number assays

**Fig. 1:**
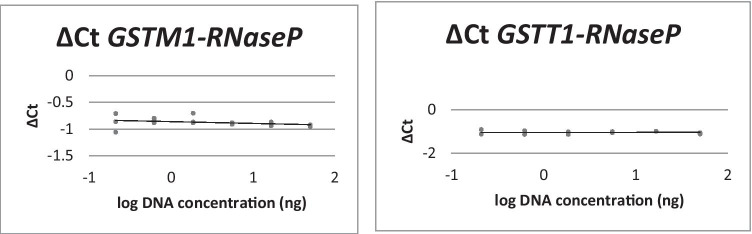
Amplification efficiencies of GST genes and RNaseP in duplex reactions. The ΔCt values are plotted against log DNA concentration, showing a slope of − 0.03 for *GSTM1 *(*left*) and *RNaseP* and a slope of − 0.005 for *GSTT1* and *RnaseP *(*right*), meeting the requirements of using the ΔΔCt method in both duplex reactions

Comparison of the amplification efficiencies of the GSTs and *RNaseP* in duplex reactions resulted in an amplification efficiency of almost 100%, the efficiencies for *GSTM1* and *RNaseP* being 99.14% and 99.77%. The efficiencies for *GSTT1* and *RNaseP* were 100.92% and 100.45%. The corresponding curves, in which the ΔCt values were plotted against log DNA concentration to compare these efficiencies, show a slope of − 0.03 for *GSTM1* and *RNaseP* and a slope of − 0.005 for *GSTT1* and *RNaseP* (Fig. 1), which meets the requirements for the use of the ΔΔCt method in both cases.

The difference in ΔΔCt values between 1/1 and 1/0 was − 0.8 for *GSTM1* and − 0.86 for *GSTT1*. For 2/1 vs. 1/0 the differences in the ΔΔCt values were − 1,33 for *GSTM1* and − 1.48 for *GSTT*. Table 1 shows the mean Ct, ΔCt, and ΔΔCt values for *GSTM1* and *GSTT1*, respectively.

**Table 1:** Mean of Ct, ∆Ct, und ∆∆Ct values with their standard deviation (SD) for *GSTM1* und *GSTT1*

Number of existing gene copies in chromosome set		Mean Ct (SD)	Mean ∆Ct (SD)	Mean ∆∆Ct (SD)
GSTM1	1/0	25,66 (0,89)	0,38 (0,22)	0,83 (0,07)
	1/1	26,00 (1,60)	− 0,38 (0,16)	0,03 (0,08)
	2/1	27,25 (0,93)	− 1,00 (0,17)	− 0,5 (0,06)
GSTT1	1/0	25,31 (1,21)	− 0,2 (0,17)	1,00 (0,04)
	1/1	23,78 (0,78)	− 1,08 (0,19)	0,14 (0,1)
	2/1	23,55 (0,00)	− 1,72 (0,00)	− 0,48 (0,00)

**Fig. 2:**
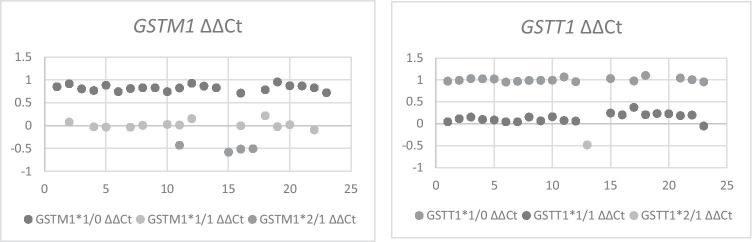
Samples (SIDS and controls) were analysed in a total of 23 individual reaction plates. Each dot represents the mean ΔΔCt value observed on each respective plate. Different shades of grey refer to groups of 1 gene copy (*1/0 genotype), 2 gene copies (*1/1 genotype), and 3 gene copies (*2/1 genotype). There are no significant differences between plates

Table 1 gives an overview of ΔΔCt values, and Fig. 2 shows that a clear and unambiguous distinction between genotypes is possible.

### CNV analysis

Copy number assessment was successful in 251 samples for *GSTM1* and 252 samples for *GSTT1.* See Table 2 for details. There was a significant difference in the frequency of gene deletions between SIDS cases and controls for the *GSTM1* gene: overall, gene deletions of *GSTM1* occurred significantly more frequently in the SIDS case group compared to the control group (Chi^2^ test; *p* < 0.01). Heterozygous deletions were significantly more common in the SIDS case group than in the control group (Chi^2^ test; *p* < 0.01). At approximately 55%, homozygous gene deletions were more common in the control group. This is surprising, since it was expected to observe more homozygous deletions in the SIDS group. It is known, however, that reduced enzyme activity due to gene deletions can be compensated by other members of the GST family, particularly in 0/0 genotypes (see below). Therefore, the effect of a homozygous gene deletion might not be as severe as expected, which might explain the abundance of 0/0 genotypes in the control group.

**Table 2:** Frequency of the *GSTM1* and *GSTT1* genotypes in SIDS cases and controls

		SIDS cases		*Controls*	
Number of existing gene copies in chromosome set		*n* = 251	%	*n* = 167	%
GSTM1	0/0	131	52,19%	92	55,09%
	1/0	93	37,05%	38	22,75%
	1/1	26	10,36%	32	19,16%
	2/1	1	0,40%	5	2,99%
		*n* = 252	%	*n* = 168	%
GSTT1	0/0	53	21,03%	31	18,45%
	1/0	127	50,40%	88	52,38%
	1/1	71	28,17%	49	29,17%
	2/1	1	0,40%	0	0,00%

The GSTT1*0/0 genotype was observed in 21.03% of the SIDS samples tested, while 50.40% showed a GSTT*1/0 genotype and 28.17% were a GSTT1*1/1 genotype. In one SIDS case (0.4%), there was a GSTT1*2/1 genotype present.

There is no significant difference between the SIDS group and the control group with regard to the gene copy distribution of *GSTT1* (Table 2).

The results on the frequency of gene deletions and the occurrence of SIDS confirm results of previous studies: Both in the SIDS case group and in the control group, there was a complete gene loss of the *GSTM1* genes in a little more than 50% of the samples examined, which is in agreement with the results of various studies that described a complete gene loss of *GSTM1* in approximately 50% of the European population [22, 28, 39]. As expected, the *GSTT1* gene also had a 0 genotype in both groups in about 20% of the samples [14, 22, 39].

Our study shows a relationship between the number of copies of *GSTM1* and the occurrence of SIDS, but this does not apply to *GSTT1*. Chen et al. discuss in their paper that neither *GSTM1* nor *GSTT1* are involved in the aetiology of SIDS [33], while Filonzi et al. [13] did observe a potential connection between the copy number of *GSTM1* and SIDS. They and Rand et al. also point out that *GSTT1* has no impact on SIDS [13, 14]. In general, gene deletions of the *GSTM1* gene occurred more frequently in the SIDS case group than in the control group, which indicates that the number of copies of *GSTM1* might play a role in the aetiology of SIDS.

With a larger number of SIDS cases, examining the combined occurrence of gene deletions within the GST gene family and their effects on the occurrence of sudden infant death would be possible in more detail. This is particularly interesting as there are indications that a GSTM1*0/0 genotype can be partially compensated for by another gene in the GST family. Fuciarelli et al. [40] found that the GSTM1*0/0 genotype had higher *GSTT1* enzyme activity; however, this could not be confirmed in the study by Saitou et al. [41]. Instead, it was suspected that *GSTM1* is more likely to be compensated by *GSTP1*, since these two enzymes have more common substrates. Bhattacharjee et al. [42] showed, for example, an overexpression of *GSTM2* in a *GSTM1* null genotype, so that GST activity in the plasma was not impaired. These were the results of a cell culture study that examined how, with regard to overexpression and compensation by *GSTM2*, the knockout of *GSTM1* affects the breakdown of the glutathione-sulforaphane conjugate, which triggers cell death. Due to the high sequence homology and overlap in the substrates, there seems to be a well-functioning compensation mechanism for the GST genes. Thus, analysing gene activity and compensation in SIDS cases should be a focus in future studies because the significantly higher abundance of *GSTM1* gene deletions in SIDS compared to controls suggests a potential malfunction of such compensation strategies.

### Genotype and lifetime

The number of *GSTM* gene copies has no significant influence on the children’s lifetime (Kruskal–Wallis test; *p* = 0.848 [*GSTM1*] and *p* = 0.329 [*GSTT1*]). Chen et al. [33] found no significant relationship between the genotype of *GSTM1*/*GSTT1* and the SIDS risk. Rand et al. also did not establish any significant connection between the *GSTT1* genotype and the SIDS risk [14]. Although these studies did not investigate the influence of the genotype on lifetime, it can nevertheless be concluded that the genotype alone neither has an influence on the point in time at which SIDS occurs, nor on the SIDS risk in general.

### Smoking behaviour

In the SIDS group, the number of victims that were exposed to cigarette smoke (*n* = 168) was significantly higher than the number of victims without cigarette smoke exposure (*n* = 35) (exact binomial test, two-sided, *p* < 0.01). Detailed information of smoking exposure is given in Table 3. In 83% of SIDS cases, at least one parent was a smoker. Overall, the fathers were heavier smokers than the mothers (Chi^2^ test; *p* < 0.01). Smoking was more common in parents of SIDS victims compared to the general population in Germany at the time of the GeSID study: 22% of woman and 36% of men above the age of 15 were smokers. Heavy smokers (> 20 cigarettes per day) accounted for 6% of the male and 2% of the female population [43].

**Table 3:** Details on cigarette smoke exposure of SIDS victims included in this study

Smoke exposure	*n *= 112	%
Mother and father are moderate smokers	60	53.6%
Mother smokes moderately, father is strong smoker	28	25.0%
Mother is strong smoker, father smokes moderately	7	6.3%
Mother and father are strong smokers	7	6.3%
Father is strong smoker, no information for mother available	1	0.9%
Mother smokes moderately, no information for father available	8	7.1%
Mother is strong smoker, no information for father available	1	0.9%
Smoking in the presence of the child	*n* = 203	%
No – non-smoker	35	17.2%
No – smoker	91	44.8%
No – unreliable	2	1.0%
Yes	32	15.8%
Yes – open window	1	0.5%
Yes – exceptionally	39	19.2%
No information available	3	1.5%

### Smoking behaviour and genotype

There was no statistical significance regarding the *GSTM1* or *GSTT1* genotype and the mother’s or father’s smoking behaviour (Chi2 test; *p* = 0.083 [*GSTM1*, mothers]; *p* = 0.657 [*GSTM1*, fathers]; *p* = 0.682 [*GSTT1*, mothers]; *p* = 0.516 [*GSTT1*, fathers]).

### Smoking behaviour and lifetime

The mean lifetime of SIDS victims whose mothers smoked was 101 days, and 154 days if the mother was a non-smoker. Thus, the mother’s smoking behaviour had an impact on the child’s lifetime (Kruskal–Wallis test; *p* = 0.024). These results coincide with results from Haglund et al. [11], who found that the risk for early SIDS (death occurring between 7 and 67 days) was increased when mothers were moderate smokers. It should be kept in mind, however, that SIDS is a complex disease and other risk factors might also influence the lifetime of the affected child.

While the number of cigarettes smoked per day and the child’s total smoke exposure influence the risk of SIDS significantly [9, 11, 31], this finding narrowly missed statistical significance (Kruskal–Wallis test; *p* = 0.052) for the exact number of cigarettes smoked by the mother and the lifetime of the child. Children of moderately smoking mothers did not live longer than children of mothers with a strong smoking habit. We did, however, observe a statistically significant difference between the lifetime of SIDS victims of non-smoking mothers and smoking mothers; i.e., mothers who were moderate (Kruskal–Wallis test; *p* = 0.013) or heavy smokers (Kruskal–Wallis test; *p* = 0.032).

If the father was a smoker, the median lifetime was 110 days, and if he was not smoking, it was 104.5 days. The relationship between the father’s smoking behaviour and the child’s lifetime is not statistically significant (Chi^2^ test; *p* = 0.912).

### Smoking behaviour, genotype, and lifetime

There was a direct significant correlation between the *GSTM1* genotype, the mother’s smoking behaviour, and the child’s lifetime (Kruskal–Wallis test; *p* = 0.041). The mean lifetime of children with a GSTM1*0/0 genotype was 110 days. However, the median lifetime was significantly reduced to 106 days in cases where the mother smoked compared to victims without smoke exposure (median lifetime: 161 days) (Kruskal–Wallis test; *p* < 0.05). The mean was 125.86 days (SD 70.7) if the mother smoked and 163.23 days (SD 94.24) if the mother was a non-smoker. Thus, children lived longer if there was a GSTM1*0/0 genotype and the mother was a non-smoker. In children with a GSTM1*1/0 genotype, the median lifetime was 168 days (mean: 182.81 days; SD: 95.03) for non-smoking mothers and 100 days (mean: 132.18 days; SD: 89.28) for smoking mothers which is also a significant relationship (Kruskal–Wallis test; *p* < 0.05).

There were no direct significant correlations between the *GSTT1* genotype, the mother’s smoking behaviour, and the child’s lifetime (Kruskal–Wallis test; *p* = 0.223).

There was also no significant connection between the father’s smoking behaviour, the genotype of *GSTM1* and *GSTT1*, and the lifetime of the children who died from SIDS (Kruskal–Wallis test; *p* = 0.720 [*GSTM1*] and *p* = 0.497 [*GSTT1*]).

Our sample set did not contain any information on the cigarette smoke exposure of individuals of the control group. Thus, a direct comparison of smoking in combination with *GSTM1* or *GSTT1* deletions is still outstanding. Our data, however, suggest a correlation between the smoking behaviour, copy number of *GSTM1*, and the timepoint of death in the SIDS group.

## Conclusion

We conclude that the gene deletions of *GSTM1* and *GSTT1* can have an impact on the development of SIDS. As Hayes et al. [16] already described in their study, *GSTM1* and *GSTT1* can possibly be understood as disease-modulating due to their protective effect against cytotoxic influences, and not so much as disease-triggering, which can also be transferred to SIDS. SIDS is a multifactorial event, in which the absence or presence of these two genes could be the deciding factor. Further studies are needed to investigate the potential malfunction of compensation strategies within the GST gene family.

## References

[1] Krous HF (2010). Sudden unexpected death in infancy and the dilemma of defining the sudden infant death syndrome. CurrPediatr Rev.

[2] Robert Koch-Institut Gesundheit in Deutschland - Gesundheitsberichterstattung des Bundes 2015. https://www.rki.de/DE/Content/Gesundheitsmonitoring/Gesundheitsberichterstattung/GesInDtld/gesundheit_in_deutschland_2015.html

[3] Moon RY, Task Force on Sudden Infant Death Syndrome, MD, FAAP (2016). SIDS and other sleep-related infant deaths: evidence base for 2016 updated recommendations for a safe infant sleeping environment. Pediatrics.

[4] Task force on infant positioning and SIDS positioning and sudden infant death syndrome (SIDS): Update (1996). Pediatrics 98:1216–1218 8951285

[5] Homepage | Safe to sleep. https://safetosleep.nichd.nih.gov/. Accessed 2 Feb 2021

[6] Filiano JJ, Kinney HC (1994). A perspective on neuropathologic findings in victims of the sudden infant death syndrome: the triple-risk model. Biol Neonate.

[7] Banderali G, Martelli A, Landi M, Moretti F, Betti F, Radaelli G, Lassandro C, Verduci E (2015). Short and long term health effects of parental tobacco smoking during pregnancy and lactation: a descriptive review. J Transl Med.

[8] Raymond EG, Cnattingius S, Kiely JL (1994). Effects of maternal age, parity, and smoking on the risk of stillbirth. BJOG Int J Obstet Gynaecol.

[9] Mitchell EA, Ford RPK, Stewart AW, Taylor BJ, Becroft DMO, Thompson JMD, Scragg R, Hassall IB, Barry DMJ, Allen EM (1993). Roberts AP Smoking and the sudden infant death syndrome. Pediatrics.

[10] Blair PS, Fleming PJ, Bensley D, Smith I, Bacon C, Taylor E, Berry J, Golding J, Tripp J (1996). Smoking and the sudden infant death syndrome: results from 1993–5 case-control study for confidential inquiry into stillbirths and deaths in infancy Confidential Enquiry into Stillbirths and Deaths Regional Coordinators and Researchers. BMJ.

[11] Haglund B (1990). Cnattingius S Cigarette smoking as a risk factor for sudden infant death syndrome: a population-based study. Am J Public Health.

[12] Schoendorf KC (1992). Kiely JL Relationship of sudden infant death syndrome to maternal smoking during and after pregnancy. Pediatrics.

[13] Filonzi L, Magnani C, Lavezzi AM, Vaghi M, Nosetti L, Nonnis Marzano F (2018). Detoxification genes polymorphisms in SIDS exposed to tobacco smoke. Gene.

[14] Rand CM, Weese-Mayer DE, Maher BS, Zhou L, Marazita ML, Berry-Kravis EM (2006). Nicotine metabolizing genes GSTT1 and CYP1A1 in sudden infant death syndrome. Am J Med Genet A.

[15] Poetsch M, Czerwinski M, Wingenfeld L, Vennemann M, Bajanowski T (2010). A common FMO3 polymorphism may amplify the effect of nicotine exposure in sudden infant death syndrome (SIDS). Int J Legal Med.

[16] Hayes JD, Strange RC (2000). Glutathione S-transferase polymorphisms and their biological consequences. PHA.

[17] Nebert DW, Vasiliou V (2004). Analysis of the glutathione S-transferase (GST) gene family. Hum Genomics.

[18] Nebert DW (1991). Role of genetics and drug metabolism in human cancer risk. Mutat Res/Fundam Mol Mech Mutagen.

[19] Thielen A, Klus H, Müller L (2008). Tobacco smoke: unraveling a controversial subject. Exp Toxicol Pathol.

[20] Hayes JD, McLellan LI (1999). Glutathione and glutathione-dependent enzymes represent a co-ordinately regulated defence against oxidative stress. Free Radic Res.

[21] Krause G, Müller M Glutathion‐S‐transferase T1 und M1 (Genotypisierung) [Biomonitoring Methods in German language, 2004]. In: The MAK‐Collection for Occupational Health and Safety. American Cancer Society:1–25

[22] Rebbeck TR (1997). Molecular epidemiology of the human glutathione S-transferase genotypes GSTM1 and GSTT1 in cancer susceptibility. Cancer Epidemiol Biomarkers Prev.

[23] Hallier E, Langhof T, Dannappel D, Leutbecher M, Schroder K, Goergens HW, Muller A, Bolt HM (1993). Polymorphism of glutathione conjugation of methyl bromide, ethylene oxide and dichloromethane in human blood: influence on the induction of sister chromatid exchanges (SCE) in lymphocytes. Arch Toxicol.

[24] Weinhold B (2009). CHILDREN'S HEALTH: gene variants may predict lung health. Environ Health Perspect.

[25] Filonzi L, Nosetti L, Magnani C, Vaghi M, Fenjiep AFN, Marzano FN (2016). ALTE and smoking exposure: which role of detoxification genes polymorphisms?. Clin Genet.

[26] Xu S, Wang Y, Roe B, Pearson WR (1998). Characterization of the Human Class Mu GlutathioneS-Transferase Gene Cluster and the GSTM1Deletion. J Biol Chem.

[27] Sprenger R, Schlagenhaufer R, Kerb R, Bruhn C, Brockmöller J, Roots I (2000). Brinkmann U Characterization of the glutathione S-transferase GSTT1 deletion: discrimination of all genotypes by polymerase chain reaction indicates a trimodular genotype-phenotype correlation. Pharmacogenetics.

[28] Seidegard J, Vorachek WR, Pero RW (1988). Pearson WR Hereditary differences in the expression of the human glutathione transferase active on trans-stilbene oxide are due to a gene deletion. Proc Natl AcadSci U S A.

[29] Bruhn C, Brockmoller J, Kerb R, Roots I, Borchert HH (1998). Concordance between enzyme activity and genotype of glutathione S-transferase theta (GSTT1). Biochem Pharmacol.

[30] Anderson HR (1997). Cook DG Passive smoking and sudden infant death syndrome: review of the epidemiological evidence. Thorax.

[31] Anderson TM, Ferres JML, Ren SY, Moon RY, Goldstein RD, Ramirez J-M, Mitchell EA (2019). Maternal smoking before and during pregnancy and the risk of sudden unexpected infant death. Pediatrics.

[32] Schwender K, Holtkötter H, Johann KS, Glaub A, Schürenkamp M, Sibbing U, Banken S, Vennemann M, Pfeiffer H, Vennemann M, GeSID Study Group (2016). Sudden infant death syndrome: exposure to cigarette smoke leads to hypomethylation upstream of the growth factor independent 1 (GFI1) gene promoter. Forensic Sci Med Pathol.

[33] Chen CL, Liu Q, Evans WE, Sander CH, Relling MV (1997). Cytochrome P450 2D6 and glutathione S-transferase genotype in sudden infant death syndrome. J Paediatr Child Health.

[34] Findeisen M, Vennemann M, Brinkmann B, Ortmann C, Rose I, Kopcke W, Jorch G, Bajanowski T (2004). German study on sudden infant death (GeSID): design, epidemiological and pathological profile. Int J Legal Med.

[35] Schumann S (2013) Sequenzanalyse des mitochondrialen Genoms beim Syndrom des Plötzlichen Säuglingstodes (SIDS), Dissertation. Westfalische Wilhelms-Universitat Munster, Munster. https://repositorium.uni-muenster.de/document/miami/c4ccfd55-b9e3-43fb-86ef-9dd23a0d541c/diss_schumann_stefanie.pdf

[36] Nørskov MS, Frikke-Schmidt R, Loft S, Tybjaerg-Hansen A (2009). High-throughput genotyping of copy number variation in glutathione S-transferases M1 and T1 using real-time PCR in 20,687 individuals. Clin Biochem.

[37] Rose-Zerilli MJ, Barton SJ, Henderson AJ, Shaheen SO, Holloway JW (2009). Copy-number variation genotyping of GSTT1 and GSTM1 gene deletions by real-time PCR. Clin Chem.

[38] Liew S-N, Lazaruk K, Wong L, Stevens J, Livak K (2005) Determining the Copy Number of Genes Using Real-Time Quantitative PCR. *Applied Biosystems* (Hrsg.). https://tools.thermofisher.com/content/sfs/posters/cms_042195.pdf. Accessed 22 10 2020

[39] Steck SE, Gammon MD, Hebert JR, Wall DE (2007). Zeisel SH GSTM1, GSTT1, GSTP1, and GSTA1 Polymorphisms and Urinary Isothiocyanate Metabolites following Broccoli Consumption in Humans1. J Nutr.

[40] Fuciarelli M, Caccuri A, de Francesca M, Ferazzoli F, Piacentini S, Porreca F (2009). Modulation of the GSTT1 activity by the GSTM1 phenotype in a sample of Italian farm-workers. Arch Toxicol.

[41] Saitou M, Ishida T (2015). Distributions of the GSTM1 and GSTT1 null genotypes worldwide are characterized by latitudinal clines. Asian Pac J Cancer Prev.

[42] Bhattacharjee P, Paul S, Banerjee M, Patra D, Banerjee P, Ghoshal N, Bandyopadhyay A, Giri AK (2013). Functional compensation of glutathione S-transferase M1 (GSTM1) null by another GST superfamily member, GSTM2. Sci Rep.

[43] Gesundheitsbericht für DeutschlandGesundheitsberichterstattung [GBE] des Bundes ; Ergebnis eines Forschungsvorhabens : health monitoring of the federation ; result of a research project = Health report for Germany. Stuttgart: Metzler-Poeschel 1998

